# Blood transcriptomic diagnosis of pulmonary and extrapulmonary tuberculosis

**DOI:** 10.1172/jci.insight.87238

**Published:** 2016-10-06

**Authors:** Jennifer K Roe, Niclas Thomas, Eliza Gil, Katharine Best, Evdokia Tsaliki, Stephen Morris‑Jones, Sian Stafford, Nandi Simpson, Karolina D Witt, Benjamin Chain, Robert F Miller, Adrian Martineau, Mahdad Noursadeghi

**Affiliations:** 1 Division of Infection and Immunity, University College London, London, United Kingdom.; 2 Department of Microbiology, University College London Hospitals NHS Trust, London, United Kingdom.; 3 Blizard Institute, Queen Mary University of London, Barts and The London School of Medicine and Dentistry, London, United Kingdom.; 4 Research Department of Infection and Population Health, University College London, London, United Kingdom.; 5 National Institute for Health Research University College London Hospitals Biomedical Research Centre, London, United Kingdom.

## Abstract

**BACKGROUND.** Novel rapid diagnostics for active tuberculosis (TB) are required to overcome the time delays and inadequate sensitivity of current microbiological tests that are critically dependent on sampling the site of disease. Multiparametric blood transcriptomic signatures of TB have been described as potential diagnostic tests. We sought to identify the best transcript candidates as host biomarkers for active TB, extend the evaluation of their specificity by comparison with other infectious diseases, and to test their performance in both pulmonary and extrapulmonary TB.

**METHODS.** Support vector machine learning, combined with feature selection, was applied to new and previously published blood transcriptional profiles in order to identify the minimal TB‑specific transcriptional signature shared by multiple patient cohorts including pulmonary and extrapulmonary TB, and individuals with and without HIV-1 coinfection.

**RESULTS.** We identified and validated elevated blood basic leucine zipper transcription factor 2 (*BATF2*) transcript levels as a single sensitive biomarker that discriminated active pulmonary and extrapulmonary TB from healthy individuals, with receiver operating characteristic (ROC) area under the curve (AUC) scores of 0.93 to 0.99 in multiple cohorts of HIV-1–negative individuals, and 0.85 in HIV-1–infected individuals. In addition, we identified and validated a potentially novel 4-gene signature comprising CD177, haptoglobin, immunoglobin J chain, and galectin 10 that discriminated active pulmonary and extrapulmonary TB from other febrile infections, giving ROC AUCs of 0.94 to 1.

**CONCLUSIONS.** Elevated blood *BATF2* transcript levels provide a sensitive biomarker that discriminates active TB from healthy individuals, and a potentially novel 4-gene transcriptional signature differentiates between active TB and other infectious diseases in individuals presenting with fever.

**FUNDING.** MRC, Wellcome Trust, Rosetrees Trust, British Lung Foundation, NIHR.

## Abstract 2

Blood BATF2 transcripts provide a single biomarker for active tuberculosis and a novel four-gene transcriptional signature differentiates active TB from other infectious diseases with fever.

## Introduction

The laboratory diagnosis of active tuberculosis (TB) is only achieved in approximately 60% of patients. This depends on microbiological identification of *Mycobacterium tuberculosis* (Mtb), commonly undermined by the need to obtain poorly accessible samples from the site of disease and by its poor sensitivity in extrapulmonary TB (1–4). The GeneXpert MTB/RIF PCR test for nucleic acid detection can give positive results within hours (5), but the fastest liquid culture systems detect bacteria in 10 to 19 days and require 6 weeks to obtain a definitively negative result, thereby delaying clinical decisions (6). Development of novel TB diagnostics is focused on rapid tests in easily obtained clinical samples (7). These aim to give better negative predictive value than current tests, particularly when clinical sampling of the site of disease is difficult and in high-transmission settings. They are also required to provide high positive predictive value in regions of low TB incidence where alternative infections are more likely, and in immunocompromised patients who are at risk of diverse infectious diseases.

Whole-blood transcriptomics has emerged ahead of proteomics and metabolomics for diagnostic biomarker discovery in TB as a result of well-established sample-processing pathways, and has led to development of rapid sample‑in-answer-out multiplex PCR platforms (8). Numerous studies have described differential gene expression signatures in patients with active pulmonary TB compared with healthy uninfected individuals and those with latent TB infection (LTBI) (9–18). There has been comparatively little assessment of blood transcriptomes in extrapulmonary TB and limited evaluation of the specificity of TB-associated blood transcriptional signatures in comparison with other infectious or inflammatory diseases. The most recent studies have sought to reduce the number of genes in a diagnostic signature, achieving as few as 3 or 4 genes to discriminate active TB from healthy individuals with or without LTBI, or to discriminate active TB from other diseases with variable accuracy (17, 18). In the present study, we sought to rank the genes that individually or in combination discriminated patients with active TB from all healthy individuals in diverse study cohorts including asymptomatic individuals with no prior TB exposure, those with LTBI, and those who have recovered from TB. We then proceeded to test the specificity of peripheral blood gene expression signatures associated with active TB by comparison with a diverse repertoire of other infectious diseases in patients presenting to hospital and extended our assessment of these diagnostic transcriptional biomarkers to HIV‑infected patients and those with extrapulmonary TB.

## Results

### Comparison of blood transcriptomes in active TB and after long-term recovery.

**Figure 1:**
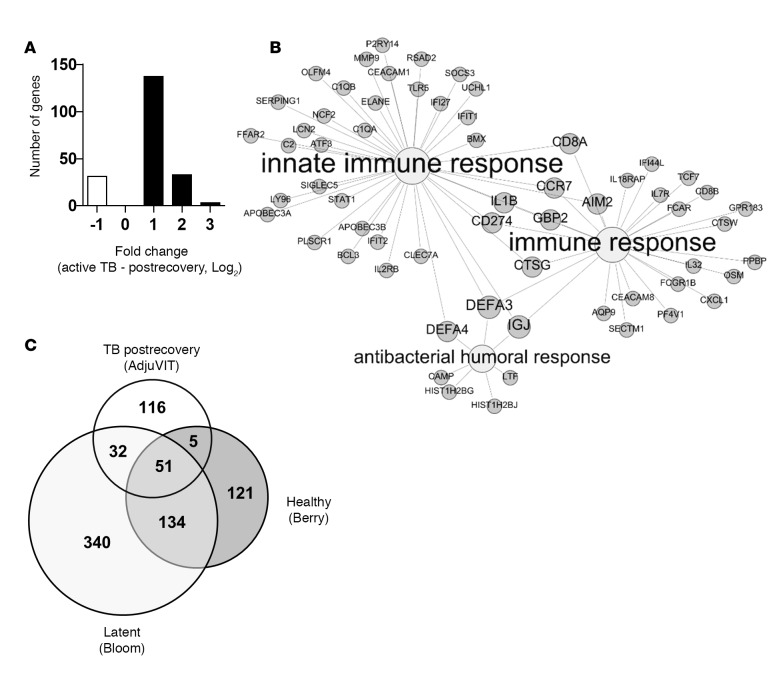
Blood transcriptional signatures associated with active tuberculosis (TB). (**A**) Statistically significant (*P* < 0.05 by Mann Whitney *U* test with false discovery rate of < 5%) greater than 2‑fold differences in transcript abundance in genome‑wide blood transcriptional profiles of patients with active TB compared with postrecovery samples in the AdjuVIT cohort. (**B**) Gene ontology analysis of the genes in **A** that were expressed at higher levels in AdjuVIT active TB samples than in the postrecovery samples. (**C**) Comparison of significant blood gene expression differences in active TB and different healthy states from 3 different studies: TB after recovery (AdjuVIT), healthy volunteers (Berry), or people with latent TB (Bloom).

We first sought to identify the peripheral blood transcriptional signature of active TB by comparison with subjects sampled from the same cohort after recovery, 2–4 years after completion of TB treatment using samples obtained from the AdjuVIT study population comprising HIV-negative patients with smear- and culture-positive pulmonary TB (Table 1) (19). This analysis revealed statistically significant and greater than 2‑fold gene expression differences in 204 unique protein-coding transcripts (Figure 1A). Like other published data, active TB in this cohort was associated with increased expression of genes associated with immune responses (Figure 1B), which showed some overlap with 2 other published blood transcriptional signatures in adult patients with active pulmonary TB compared with either healthy volunteers (10) or subjects with LTBI (11) (Figure 1C). Consistent with previous studies (10), these transcripts showed significant enrichment for components of IFN‑associated pathways (Supplemental File 2; supplemental material available online with this article; doi:10.1172/jci.insight.87238DS1).

### Application of support vector machine learning with feature selection to discriminate active TB from healthy states.

**Figure 2:**
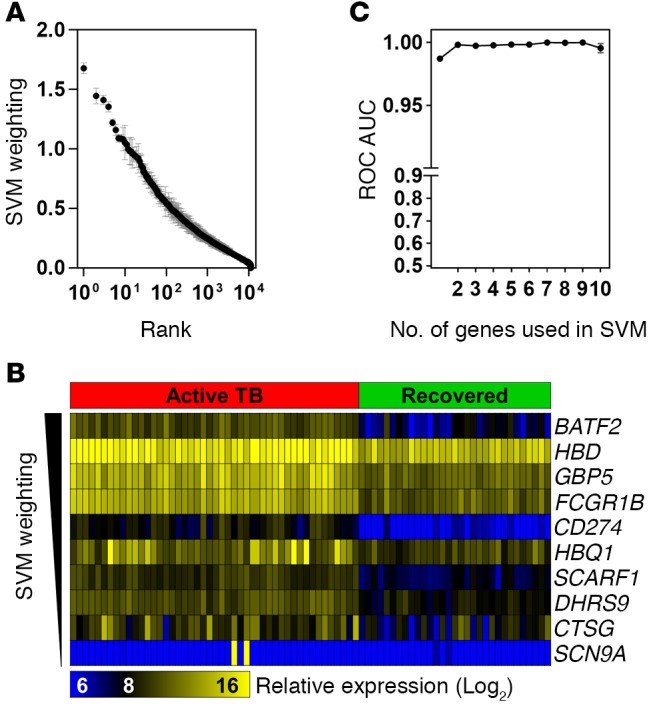
Transcriptomic classification of active tuberculosis (TB) and healthy cases using support vector machines (SVMs) to identify most discriminating features. (**A**) Rank order of weightings for each gene in the SVM training model in patients with active TB or after recovery in the AdjuVIT cohort. (**B**) Relative expression of top 10 SVM-ranked genes. (**C**) Receiver operating characteristic (ROC) area under the curve (AUC) scores of SVM classification of TB at diagnosis vs. postrecovery using a cumulative number of genes in rank order of weightings. In **A** and **C**, data points show the mean with 95% confidence intervals of SVM results obtained from 100 iterations in which the dataset was randomly split into equal training and test sets.

Our aim was to identify the fewest transcripts that may be used as a diagnostic biomarker for active TB. We used support vector machines (SVMs) to derive discriminating models in the transcriptomic data from patients with and without active TB. This approach allowed us to calculate a weighting for each dimension of the data, relative to its influence in the classification model, and thereby provided an approach to selecting the discriminating features in genome‑wide data. We trained the SVM model on multiple random samples of half the transcriptomes from the AdjuVIT cohort of active TB and postrecovery cases and ranked all the genes in order of average SVM weightings (Figure 2A). The relative expression of the highest ranked transcripts was generally higher in active TB compared with postrecovery cases (Figure 2B). We tested a cumulative number of genes in rank order of their average weightings for their ability to discriminate between active TB and postrecovery cases using data from half the samples to train the model, and the remaining half of the data to test the classification accuracy. In order to mitigate against sampling error, we performed 100 random train/test sequences to give average receiver operating characteristic (ROC) area under the curve (AUC) scores (Figure 2C). Remarkably, this analysis showed that the highest ranked transcript alone consistently achieved ROC AUC scores of greater than 0.95. This transcript represented expression of the IFN-inducible gene for basic leucine zipper transcription factor 2 (*BATF2*), which was consistently the most highly ranked feature of the data in all the SVM models derived from multiple subsamples (Figure 2A).

### BATF2 discriminates active TB from healthy states in multiple study cohorts.

**Figure 3:**
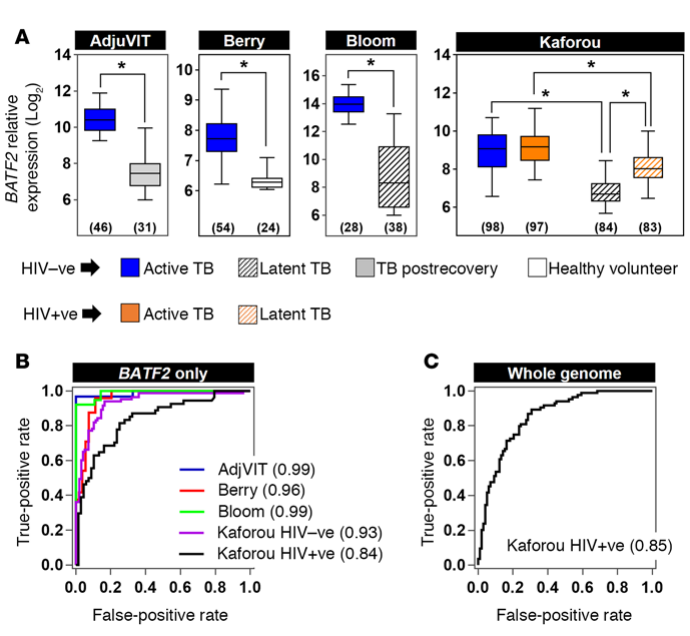
Classification of active tuberculosis (TB) and healthy cases using blood *BATF2* transcript expression levels. (**A**) Relative *BATF2* gene expression in blood samples from separate HIV-negative and HIV-positive patient cohorts comparing active TB with either postrecovery patients (AdjuVIT), LTBI (Bloom and Kaforou), or healthy volunteers (Berry). Box and whisker plots represent median, interquartile, and full range of data points. Number of data points in each group is shown in parentheses below each plot. *P < 0.0001 (Mann-Whitney *U* test). (**B**) Receiver operating characteristic (ROC) analyses for discrimination of active TB in each of these cohorts using blood levels of *BATF2* expression only. (**C**) ROC performance of SVM discrimination of active TB from LTBI in HIV-positive patients using genome‑wide blood transcriptional profiles after training on patients with active TB and after recovery. In **B** and **C**, ROC AUCs are shown in parentheses for each cohort.

Having identified peripheral blood *BATF2* transcript levels as a biomarker for active TB in the AdjuVIT cohort, we sought to test its ability to discriminate active TB from healthy states in multiple independent cohorts. *BATF2* expression in patients with active TB was significantly higher than that of healthy volunteers (Berry cohort) (10) and patients with LTBI (Bloom and Kaforou cohorts) (11, 15), irrespective of HIV status, representing data from 402 patients in total (Figure 3A). Among HIV-negative patients in these studies, peripheral blood *BATF2* expression discriminated between active TB and the various healthy cases described in each cohort with ROC AUC scores of 0.93 to 0.99 (Figure 3B). *BATF2* levels discriminated less well between active TB and LTBI cases amongst HIV-infected patients in the Kaforou cohort (ROC AUC of 0.84). In this cohort, high *BATF2* expression in patients with active TB was not significantly affected by HIV coinfection, but LTBI cases with HIV coinfection had significantly higher *BATF2* levels than HIV-negative cases (Figure 3A), partially confounding accurate discrimination between active and latent TB in HIV-infected patients by this measurement. An SVM model trained using genome‑wide data from the AdjuVIT trial to discriminate active TB and postrecovery cases also achieved a ROC AUC of 0.85 for classification of active and latent TB among HIV-infected patients from the Kaforou cohort (Figure 3C). This analysis suggests that in the context of HIV‑1 infection, inclusion of additional parameters, even up to a genome‑wide level, may not achieve substantially better classification accuracy than *BATF2* alone, although the use of alternative kernel functions in the SVM model that may improve classification accuracy were not evaluated in the present study (20). Of note, the original description of the Kaforou cohort identified a 27-gene signature that discriminated active TB and LTBI in HIV-infected subjects with a ROC AUC of 0.97 (15).

Two other reports have recently proposed the application of a 4-gene transcriptional signature comprising *GBP1*, *ID3*, *P2RY14*, and *IFITM3* (17), and a separate 3-gene transcriptional signature comprising *GBP5*, *DUSP3*, and *KLF2* (18), as the basis of potential diagnostic tests for active TB. In order to compare their performance with that of *BATF2* alone, we trained SVM models based on each signature using the AdjuVIT active TB and postrecovery samples and tested their classification accuracy in the Berry, Bloom, and Kaforou datasets (Table 2). The ROC AUC scores for each signature ranged from 0.91 to 0.99 in HIV-negative cases, and was 0.88 in HIV-positive cases. The same HIV-positive cases in the original reports of these alternative gene signatures achieved a ROC AUC of 0.84 using random forest models with *GBP1*, *ID3*, *P2RY14*, and *IFITM3* (17), and a ROC AUC 0.89 using the difference of means of *GBP5*, *DUSP3*, and *KLF2* (18). Therefore, we concluded that these 2 alternative multiparameter transcriptional signatures were also partially confounded by HIV infection and achieved only modestly better performance than *BATF2* used as a single biomarker.

**Figure 4:**
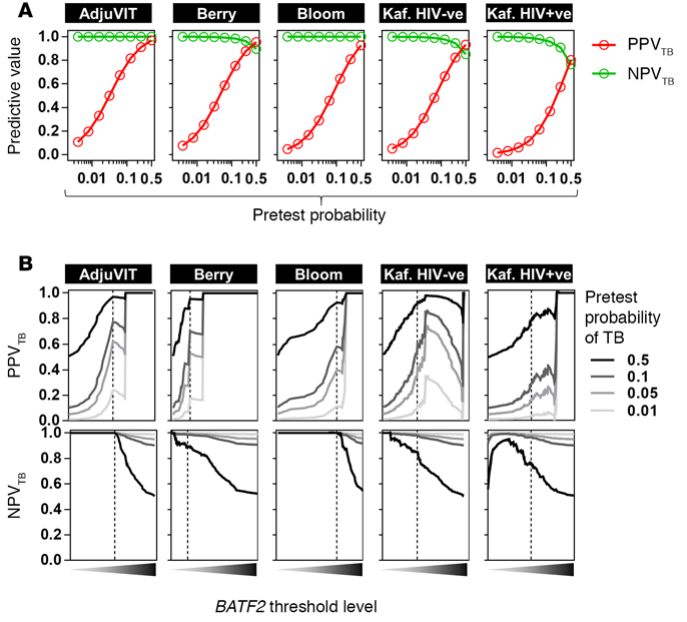
Positive and negative predictive value of blood *BATF2* transcript levels to discriminate active tuberculosis (TB) from healthy states in multiple cohorts. The positive predictive value (PPV_TB_) and negative predictive value (NPV_TB_) to discriminate active TB cases from different healthy states is shown for each study cohort for pretest probabilities of active TB ranging from 1% to 50% using the optimal *BATF2* levels determined by the Youden index of receiver operating characteristic curves in Figure 3 (**A**), and for a range of blood *BATF2* threshold levels in which the dotted lines represent the *BATF2* threshold levels derived from the Youden index (**B**).

The likelihood of any test giving a true result (predictive value) is a function of the pretest probability of the diagnosis combined with the false-positive and false-negative error rates. Bayes’ theorem of conditional probability is widely used to estimate the positive predictive value (PPV) and negative predictive value (NPV) of a test (21). In the different study cohorts described above, we used this calculation to evaluate the PPV and NPV of blood *BATF2* transcript levels for identifying active TB cases from different healthy states for pretest probabilities of active TB ranging from 1% to 50% in order to reflect contexts encompassing low and high incidence of active TB (Figure 4A). At the optimal threshold of *BATF2* transcript levels, defined by the ROC curve Youden index (22), the PPV depended strongly on the assumed pretest probability. Across the full range of pretest probabilities of 1% to 50%, the NPV ranged from 85% to 100% in HIV-negative cases and 76% to 100% in HIV-positive cases. Restricting the analysis to pretest probabilities of 1% to 10% generated NPVs of 97% to 100%, irrespective of HIV status. Different *BATF2* threshold levels could be used to achieve optimal PPV or NPV depending on the pretest probabilities (Figure 4B).

### SVM classification of active TB by comparison with other febrile illnesses.

**Figure 5:**
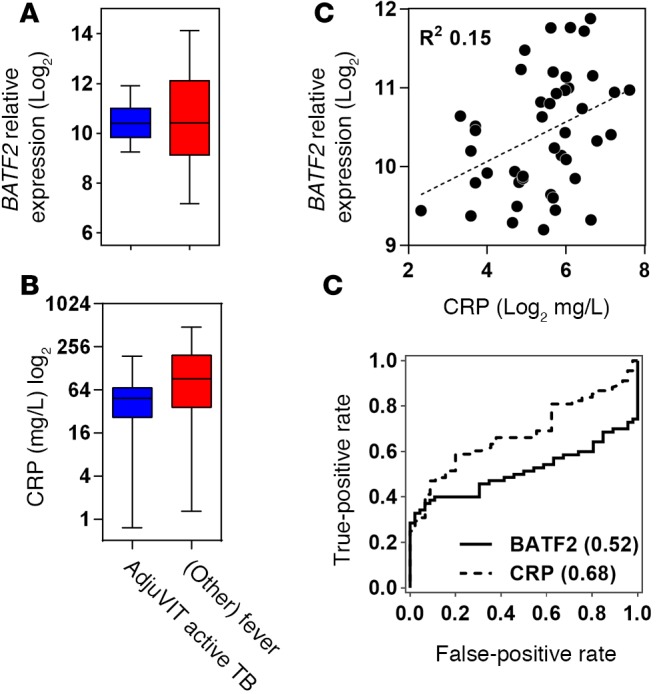
*BATF2* and C-reactive protein classification of active tuberculosis (TB) and other Fever cohort cases. (**A**) Relative *BATF2* gene expression and (**B**) serum C‑reactive protein (CRP) levels in blood samples from patients with active TB (*n* = 46) in the AdjuVIT cohort compared with patients with a spectrum of other infectious diseases presenting to hospital with fever (*n* = 70). Box and whisker plots represent median, interquartile, and full range of data points. (**C**) Scatter plot of blood *BATF2* and serum CRP in patients with active TB (AdjuVIT cohort). (**D**) Receiver operating characteristic (ROC) analyses for discrimination of active TB from other Fever cases using either blood levels of *BATF2* gene expression or serum CRP only. ROC AUCs are shown in parentheses for each cohort.

Elevated blood *BATF2* expression in HIV-infected patients with LTBI suggested that increased levels of *BATF2* may not be specific to active TB. Therefore, we compared *BATF2* expression levels in the blood transcriptomes of the AdjuVIT cohort active TB cases to those of patients presenting to hospital with febrile illnesses (Fever cohort), representing a diverse spectrum of non‑TB infectious diseases (Table 3). *BATF2* levels among Fever cohort samples showed a wide range that overlapped with those of active TB cases (Figure 5A). Therefore, we inferred that higher levels of *BATF2* may reflect nonspecific host responses to infection, much like the acute-phase reactant, serum C‑reactive protein (CRP) that is widely used as a biomarker for infection and was also not significantly different between the 2 groups (Figure 5B). *BATF2* and CRP levels among these patients showed a relatively poor correlation coefficient, suggesting that the 2 parameters were not coregulated (Figure 5C). As expected, neither CRP nor *BATF2* levels in peripheral blood were able to discriminate between active TB and Fever cohort samples (Figure 5D).

**Figure 6:**
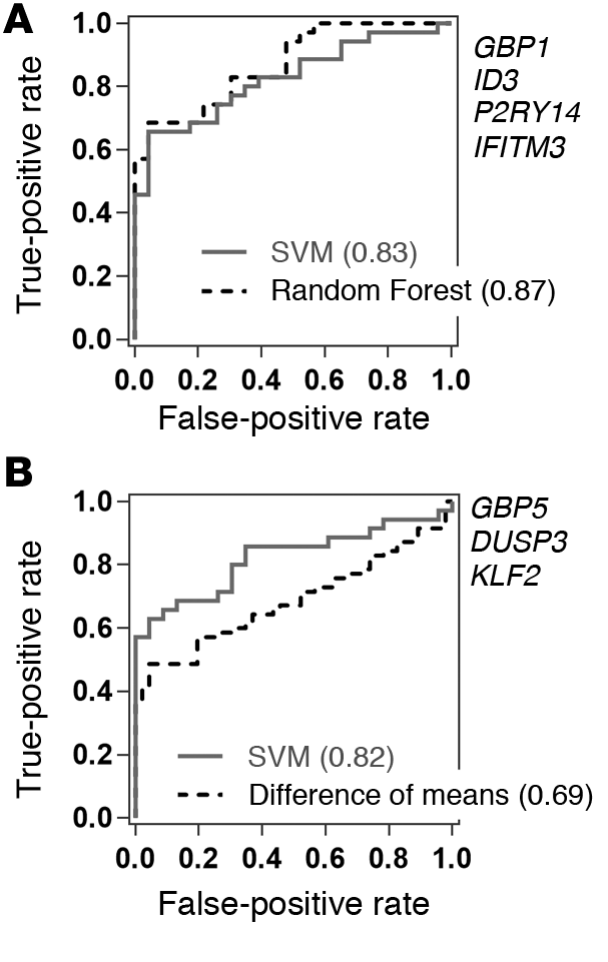
Transcriptomic classification of active tuberculosis (TB) and other Fever cohort cases. Receiver operating characteristic (ROC) analyses for discrimination of active TB from other Fever cases using expression data for (**A**) *GBP1*, *ID3*, *P2RY14*, and *IFITM3* or (**B**) *GBP5*, *DUSP3*, and *KLF2* by training SVM or random forest models as indicated on half of the AdjuVIT active TB and Fever cohort samples and testing the model performance on the second half, or by using the difference of means of (*GBP5* + *DUSP3*) – *KLF2* as described by Sweeney et al. (18). ROC AUCs are shown in parentheses for each analysis.

The alternative 4-gene and 3-gene transcriptional signatures for active TB (Table 2) were also reported to show good specificity for TB compared with other diseases (17, 18). Using SVM models trained on half the AdjuVIT active TB and Fever cohort cases and tested on the second half of data blinded to the training, we showed that each of these alternative signatures performed substantially better than *BATF2* in discriminating between active TB and other infectious diseases in the Fever cohort, achieving ROC AUCs of 0.82 to 0.83 (Figure 6A). Similar results were achieved with *GBP1*, *ID3*, *P2RY14*, and *IFITM3* using random forest models that were utilized in the original report of this signature (17) (Figure 6B). Interestingly, the difference of means of *GBP5*, *DUSP3*, and *KLF2* advocated in the original report of this signature (18) only achieved a ROC AUC of 0.69 (Figure 6B). Despite performing better than *BATF2* for discriminating between active TB and Fever cases, these ROC AUCs were significantly lower than those achieved by these signatures in comparing active TB with healthy states.

**Figure 7:**
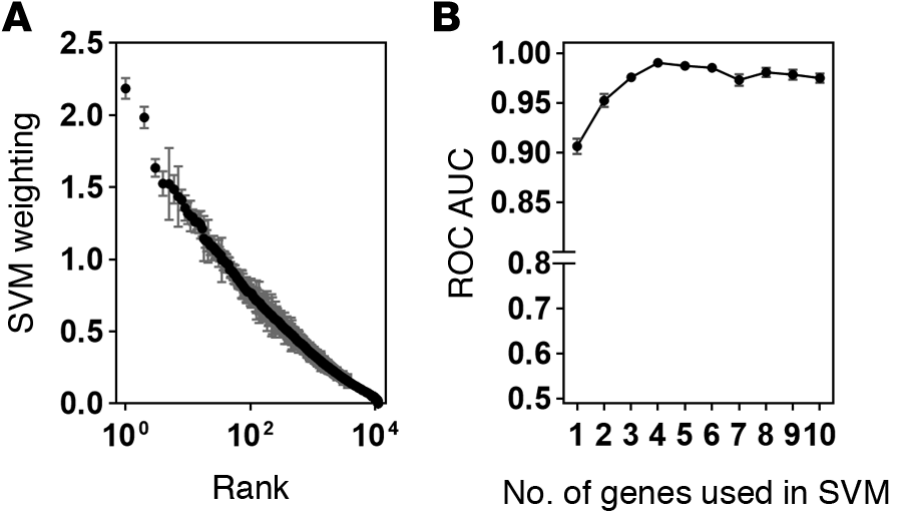
Transcriptomic classification of active tuberculosis (TB) and other infections represented by the Fever cohort cases using support vector machines (SVMs) to identify most discriminating features. (**A**) Rank order of weightings for each gene in the SVM training model of patients with active TB or other Fever. (**B**) Receiver operating characteristic (ROC) AUCs of SVM classification of active TB or other Fever using a cumulative number of genes in rank order of weightings. Data points show mean with 95% confidence intervals obtained from 100 iterations in which one half of the dataset was randomly separated into training and test sets for the SVM.

**Figure 8:**
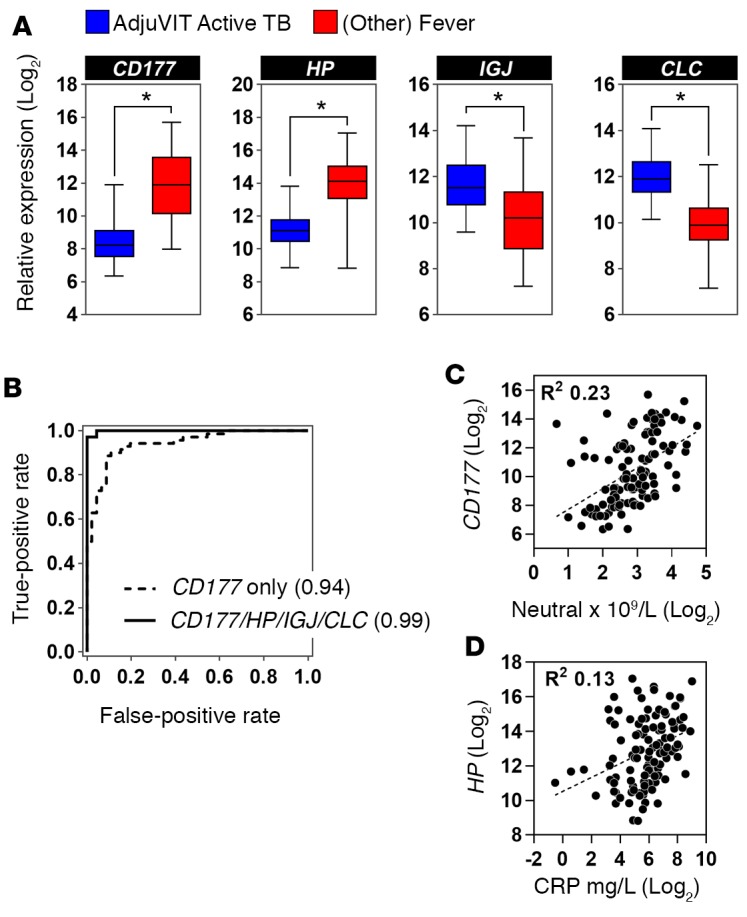
Transcriptomic classification of active tuberculosis (TB) and other Fever cohort cases with 4 genes. (**A**) Relative expression of each of the genes indicated in peripheral blood of patients with active TB (AdjuVIT, *n* = 46) and other Fever (*n* = 70) cohorts. Box and whisker plots represent median, interquartile, and full range of data points, *P < 0.0001 (Mann-Whitney *U* test). (**B**) Receiver operating characteristic (ROC) analyses of support vector machine (SVM) discrimination of active TB (AdjuVIT) from other Fever patients using expression levels of the *CD177* gene alone or all 4 of the genes indicated, by training half of the data used to derive rank order of SVM weightings and then testing on the second half of the data. ROC AUCs are shown in parentheses for each test. (**C**) Scatter plots of blood *CD177* and blood neutrophil counts, and (**D**) blood haptoglobin (*HP*) transcripts and serum C-reactive protein (CRP) concentrations in AdjuVIT active TB and Fever cohorts. In **C** and **D**, dotted line represents regression line giving R^2^ value indicated.

Therefore, we sought to further improve the classification accuracy by identifying novel peripheral blood transcripts that best differentiate active TB from other infections represented in the Fever cohort. We used half the combined AdjuVIT active TB and Fever cohort transcriptional data sets for SVM training on multiple random subsamples to identify their rank order of average weightings for discriminating active TB from Fever cases (Figure 7A). Then we tested a cumulative number of genes in rank order of their weightings for their ability to discriminate between active TB and Fever cohort cases for their classification accuracy. We carried out 100 random train/test sequences in each case using the other half of the data, and then calculated average ROC AUC scores. AUC scores increased from approximately 0.9 using the top-ranked gene alone, to 0.99 using the top 4 genes together, with no significant further gains by inclusion of additional genes (Figure 7B). Each of the 4 genes in this discriminating signature showed significantly different blood transcript levels in active TB compared with Fever cohort samples (Figure 8A). *CD177* and haptoglobin (*HP*) were expressed at higher levels in the Fever cohort samples, whereas immunoglobulin J region (*IGJ*) and galectin 10 (*CLC*) were expressed at higher levels in the active TB samples.

In order to validate their potential to discriminate between active TB and Fever cases, an SVM model was trained with transcriptional data for these 4 genes using the entire first half of AdjuVIT active TB and Fever cohort cases, and tested on the entire second half of the cases that had not been included in the identification of these genes (Figure 8B). The 4-gene signature provided almost perfect classification of the test samples with a ROC AUC of 0.99. For comparison, we also tested the top-ranked gene, *CD177*, which discriminated between active TB and Fever cohort test cases with a ROC AUC of 0.94. CD177 is a cell surface glycoprotein expressed by subpopulations of neutrophils (23–25), but the correlation coefficient with neutrophil counts in the AdjuVIT active TB and Fever cohort samples was only 0.23 (Figure 8C). This suggested that increased levels of *CD177* in the Fever cohort samples are not simply a surrogate for increased frequency of circulating neutrophils, but may represent transcriptional upregulation of *CD177* in these cases compared with active TB. We also noted that HP is recognized as an acute-phase reactant and tested the correlation of *HP* transcript levels with levels of circulating CRP, but found very modest correlation between these 2 parameters as well (Figure 8D). These data suggest that increased levels of *HP* transcripts in non‑TB infectious diseases cases reflect context-specific transcriptional upregulation rather than a surrogate for nonspecific acute-phase responses.

**Figure 9:**
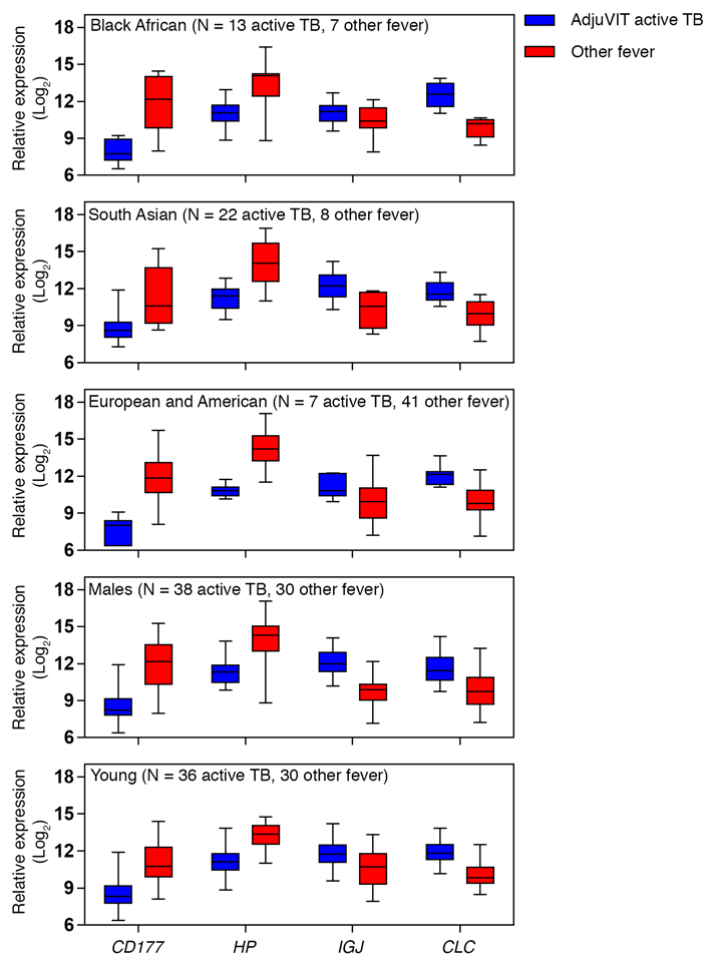
Comparison of selected biomarkers in subgroups of active tuberculosis (TB) and Fever cohort cases. Relative expression of each of the genes indicated in peripheral blood of patients of specific ethnicity, males, or age less than 40 years, within AdjuVIT active TB and other Fever cohorts. Box and whisker plots represent median, interquartile, and full range of data points. Number of cases in each group is shown in parentheses.

**Figure 10:**
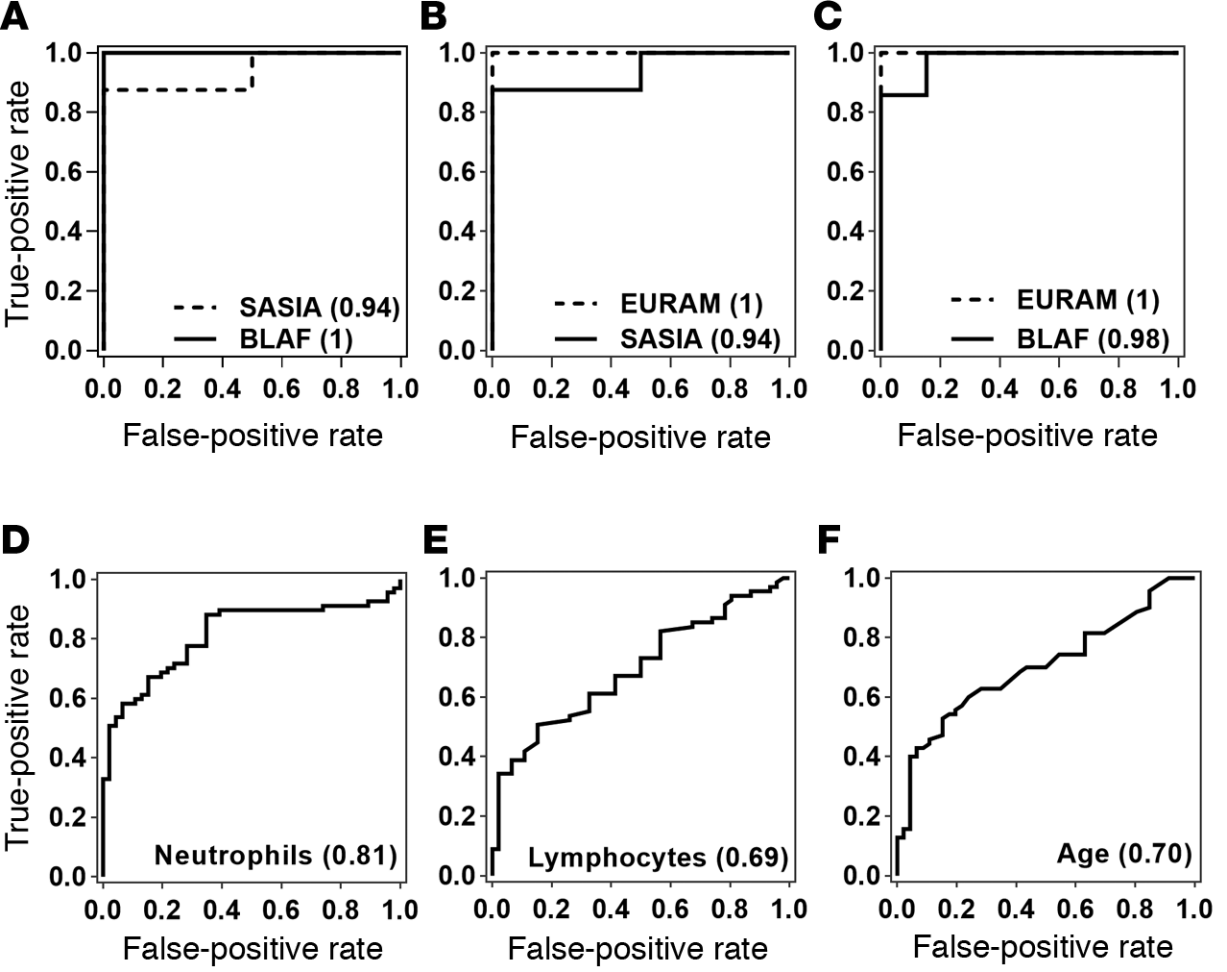
Classification of active tuberculosis (TB) and Fever cohort cases using a 4-gene transcriptional signature in specific ethnic groups, blood cell counts, and age. (**A**–**C**) Receiver operating characteristic (ROC) analyses of support vector machine discrimination of active TB (AdjuVIT) from other Fever patients using expression levels of *CD177*, *HP*, *IGJ*, and *CLC* in each of the ethnicities indicated, by training on (**A**) European and American (EURAM) patients, (**B**) Black African (BLAF) patients, and (**C**) South Asian (SASIA) patients in each cohort. (**D**–**F**) ROC analyses for discrimination of active TB from other Fever cases using either blood neutrophil counts (**D**), lymphocyte counts (**E**), or age (**F**). ROC AUCs are shown in parentheses for each test.

There were significant differences in ethnicity, age, and sex between the AdjuVIT active TB and Fever cohort patients (Supplemental Table 2). However, the different patterns of gene expression that discriminate between the 2 cohorts were evident in all ethnic groups and not confounded by age or sex (Figure 9). Moreover, training the 4-gene-signature SVM model using data from each ethnic group allowed accurate classification of cases in all other ethnic groups (Figure 10, A–C). In addition, significant differences in age and blood neutrophil or lymphocyte counts (Supplemental Table 2) discriminated poorly between active TB and Fever cases (Figure 10, D–F).

### Derivation of a single risk score for test cases.

**Figure 11:**
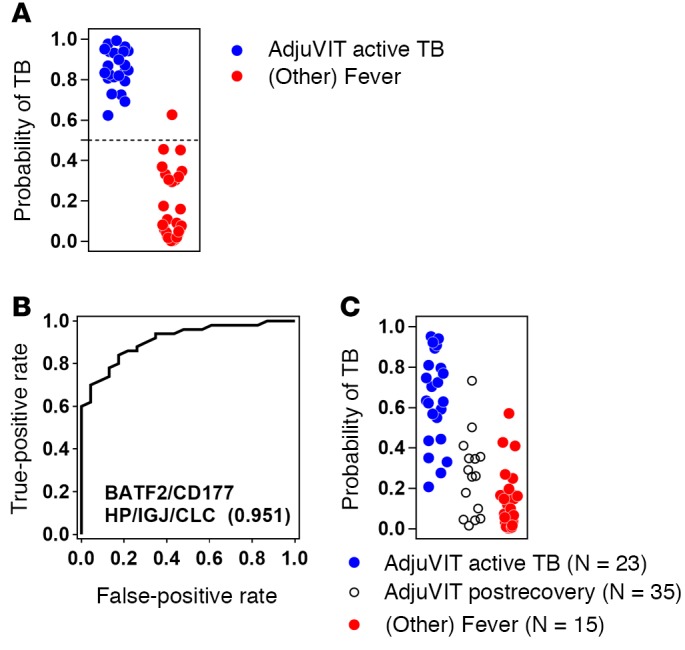
Derivation of a single risk score from multiparametric classification of active tuberculosis (TB). (**A**) Transformation of the distance of each of the test cases of active TB (AdjuVIT cohort, *n* = 23) and other infectious diseases (Fever cohort, *n* = 35) from the SVM separating hyperplane derived from the training half using *CD177*/*HP*/*IGJ*/*CLC* transcript data, to give a case-by-case probability of TB. (**B**) Receiver operating characteristic (ROC) analyses of support vector machine (SVM) discrimination of AdjuVIT active TB from pooled AdjuVIT after recovery and Fever cohort patients using expression levels of the 5 genes indicated by training half of the data and then testing on the second half of the data independently of the original derivation of the gene signature (Figure 7). ROC AUCs are shown in parentheses for each test. (**C**) Transformation of the distance of each test case in **B** from the SVM separating hyperplane derived from the training half, using all 5 genes indicated, to give a case-by-case probability of TB.

Binary classification of cases by SVM does not provide a case-by-case estimate of confidence in the accuracy of the classification. In order to achieve this, we fitted the distance of each test case from the SVM separating hyperplane to a sigmoid logistic regression function to give a probability estimate between 0 and 1 (26), thereby generating a risk score for each of the test cases based on the SVM model derived from our 4-gene signature (Figure 11A). Given that *BATF2* can discriminate between active TB and healthy cases, and that an additional 4 genes can discriminate active TB from a wide range of other infectious diseases presenting with fever, we sought to combine the expression levels of *BATF2* with *CD177*, *HP*, *IGJ*, and *CLC* in a single SVM model to discriminate active TB from postrecovery cases in the AdjuVIT cohort, and from other diseases in the Fever cohort. The SVM model was trained using the 5-gene signature on half of the AdjuVIT active TB cases in 1 group and half of the AdjuVIT postrecovery TB cases pooled with half of the Fever cohort cases in a second group. This model was then used to classify the remaining second half of the cases in all 3 groups, providing a single risk score of active TB for each case and giving a ROC AUC of 0.95 (Figure 11, B and C).

**Figure 12:**
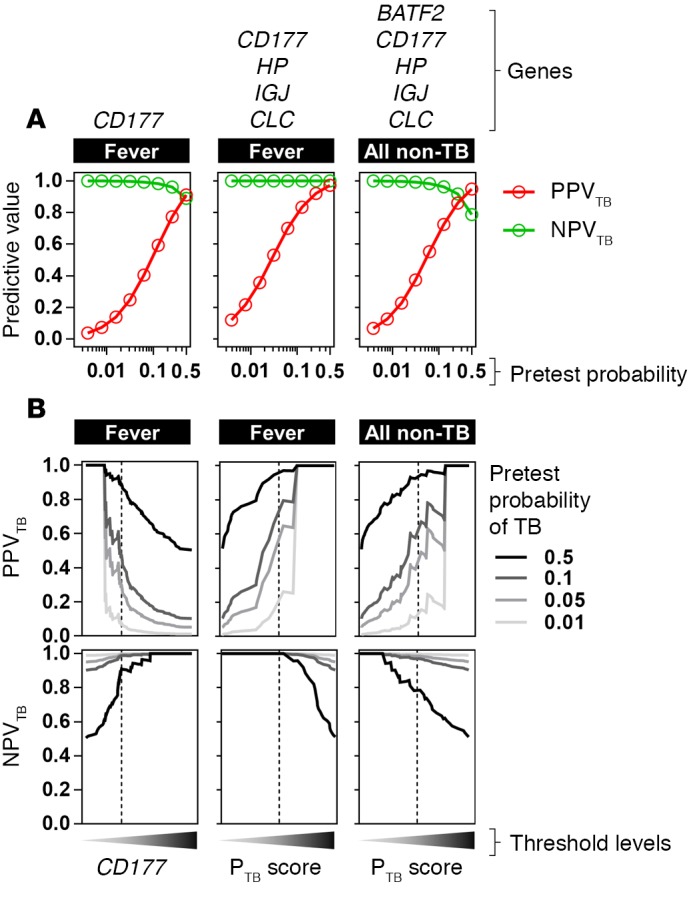
Positive and negative predictive value of selected blood transcript levels to discriminate active tuberculosis (TB) from other infectious diseases presenting with fever. The positive predictive value (PPV_TB_) and negative predictive value (NPV_TB_) to discriminate active TB cases from other infectious diseases with fever is shown for pretest probabilities of active TB ranging from 1% to 50% using the optimal levels of *CD177* alone, or the 4- and 5-gene signatures indicated, determined by the Youden index of receiver operating characteristic curves in Figures 8B and 11B (**A**), and for a range of biomarker thresholds, in which the dotted lines represent the thresholds derived from the Youden index (**B**).

Bayes’ theorem was also used to calculate the PPV and NPV of using *CD177* alone or the combination of *CD177*, *HP*, *IGJ*, and *CLC* to discriminate active TB from other infections, and the combination of *BATF2*, *CD177*, *HP*, *IGJ*, and *CLC* to discriminate active TB from either healthy states or other infections for a range of pretest probabilities, revealing the optimal PPV or NPV that can be achieved (Figure 12). At the optimal Youden index threshold, the application of the potentially novel 4-gene signature to discriminate active TB from other infectious diseases, generated wide-ranging PPVs dependent on the pretest probability but an NPV of 100%, even assuming the highest pretest probability for TB of 50%.

### Blood transcriptional signatures for active TB are independent of the site of disease.

**Figure 13:**
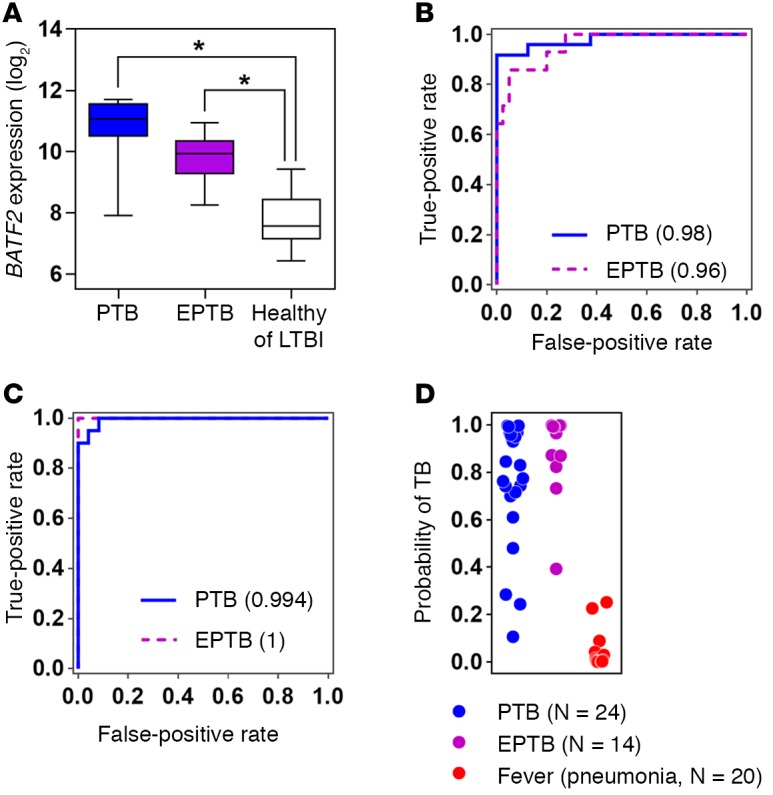
Pulmonary and extrapulmonary tuberculosis (TB) discrimination from healthy cases and non‑TB pneumonia. (**A**) Relative *BATF2* gene expression in blood samples from a new independent cohort of patients with pulmonary TB (PTB, *n* = 24), extrapulmonary TB (EPTB, *n* = 14), or from healthy volunteers (*n* = 29) and subjects with latent TB infection (LTBI, *n* = 11). Box and whisker plots represent median, interquartile, and full range of data points. *P < 0.0001 (Mann-Whitney *U* test). (**B**) Receiver operating characteristic (ROC) analyses of support vector machine (SVM) discrimination of PTB and EPTB from healthy volunteers or LTBI cases based on the *BATF2* levels in **A**. (**C**) ROC analyses of SVM discrimination of new PTB and EPTB from cohort of new non‑TB pneumonia cases after training the SVM model on active TB (AdjuVIT) and other Fever cases using relative expression levels of *CD177*/*HP*/*IGJ*/*CLC*. (**D**) Transformation of the distance of each test case in **C** from the SVM separating hyperplane, using all 4 genes indicated, to give a case-by-case probability of TB.

All the active TB cases in the AdjuVIT, Berry, and Bloom cohorts were of pulmonary TB. Novel diagnostic biomarkers for TB are particularly needed for extrapulmonary TB in which existing microbiological diagnostics have the lowest sensitivity. Therefore, we obtained new blood transcriptomic data from additional independent cases to evaluate the utility of *BATF2* and the 4-gene TB‑specific transcriptional signature described above by comparison of healthy individuals and those with LTBI, active pulmonary or extrapulmonary TB, and non‑TB respiratory tract infection before any antibiotic treatment. By comparison with healthy volunteers, blood *BATF2* transcript levels were significantly higher in both pulmonary and extrapulmonary TB cases (Figure 13A). Elevated *BATF2* classified cases of pulmonary TB with a ROC AUC of 0.98 and extrapulmonary cases with a ROC AUC of 0.96 (Figure 13B). Finally, an SVM model trained with data from AdjuVIT active TB and all Fever cohort cases using the 4-gene TB-specific signature of *CD177*, *HP*, *IGJ*, and *CLC*, discriminated between new pulmonary or extrapulmonary TB cases and non‑TB febrile pneumonia with ROC AUCs of 0.99 and 1, respectively (Figure 13, C and D). Therefore, we concluded that *BATF2* and the 4-gene TB blood transcriptional signatures performed equally well in classification of both pulmonary and extrapulmonary TB.

## Discussion

We have identified and validated elevated *BATF2* levels as a discriminating blood transcriptional biomarker for active TB compared with healthy uninfected individuals, people with LTBI, and people with long-term recovery from active TB. Our assessment of 226 cases of TB and 176 healthy individuals among HIV-seronegative adults across 4 separate study cohorts with diverse ethnic backgrounds consistently gave ROC AUCs of 0.93 to 0.99. The performance of *BATF2* as single transcriptional biomarker in these cohorts was comparable to the performance of other recently reported transcriptional signatures of active TB based on 4 genes (*GBP1*, *ID3*, *P2RY14*, and *IFITM3*) (17) or 3 genes (*GBP5*, *DUSP3*, and *KLF2*) (18).

BATF2 belongs to the activator protein 1 (AP‑1) transcription factor family, with IFN‑inducible expression in mononuclear phagocytic cells, and is upregulated by innate immune stimulation with lipopolysaccharide or Mtb. BATF2 interacts with IFN regulatory factor 1 (IRF1) to mediate downstream proinflammatory responses, some of which are also recognized as components of the host response to Mtb (27, 28). Given that systemic IFN activity is widely recognized in active TB (10), increased *BATF2* expression is most likely due to IFN responses rather than direct Mtb stimulation of circulating blood cells. Discrimination of active TB and LTBI in HIV-infected individuals using blood *BATF2* transcript levels achieved a ROC AUC level of 0.84. *BATF2* transcript levels in HIV-infected and uninfected active TB cases were not significantly different, and the variance of *BATF2* in all study groups was broadly similar. Instead, reduced classification accuracy was due to higher levels of *BATF2* in HIV-infected LTBI cases, which we speculate reflects chronic HIV-associated IFN activity (29). Longitudinal comparison of *BATF2* transcript levels in HIV‑1–infected patients before and after initiation of HIV treatment and completion of TB treatment may offer the opportunity to dissect the independent effects of HIV and TB infection on *BATF2* levels.

Our current data suggest that in HIV-infected cases, *BATF2* transcript levels may be expected to offer better negative predictive value as a biomarker for active TB than positive predictive value. Interestingly, discrimination of active TB and LTBI cases in HIV-infected people using genome‑wide transcriptional data did not yield a better ROC AUC than *BATF2* by itself, suggesting that even combinations of other transcripts will not afford better classification accuracy using SVM. This hypothesis is partially borne out by the assessment of the alternative concise transcriptional signatures described above which achieved a ROC AUC of 0.88 in the same analysis, but more complex transcriptional signatures of 27 genes were reported to achieve a ROC AUC of 0.97 to discriminate between active TB and LTBI in HIV-positive adults (15). Even in the context of HIV infection, at the optimal balance between sensitivity and specificity in ROC curve analysis, the negative predictive value of *BATF2* as a biomarker for active TB only falls below 95% if the pretest probability of active TB is greater than 15%. In settings with high pretest probability (up to 50%), alternative threshold *BATF2* levels can yield greater than 95% negative predictive value while retaining a positive predictive value of 80%. These provide better risk stratification of active TB in HIV-infected patients than any other single biomarker described to date.

Consistent with our observations in HIV‑1 infection, we found that elevated *BATF2* levels were not specific to active TB, but were also variably elevated in HIV-negative patients presenting to hospital with diverse infectious diseases. Like other nonspecific markers of inflammation such as serum CRP, they did not discriminate TB from other infections. Instead we identified and validated a separate transcriptional signature comprising 4 genes that can be summarized into a single probability score to discriminate these classes with ROC AUCs of 0.96 to 1, in analyses including 84 patients with active TB and 90 patients with other infections. This transcriptional signature provided much better discrimination of TB from the spectrum of infectious diseases included than other recently reported blood gene signatures (17, 18). In addition, we showed that the transcriptional signature was independent of multiple potential confounders such as sex, age, and ethnicity for which there were significant differences between the 2 groups. Within this 4-gene signature, transcript levels for a neutrophil G protein–linked surface glycoprotein (*CD177*), and the acute-phase hemoglobin scavenging plasma protein (*HP*), were elevated in non‑TB infection cases. By contrast, active TB cases showed elevated transcript levels for *IGJ* and the glycan-binding protein, galectin 10 (*CLC*). Given the association between CD177 and neutrophils, and that blood neutrophil counts in active TB were significantly lower than the non‑TB infection group, lower *CD177* transcript levels in active TB might also have been expected, but the correlation coefficient between these variables was relatively low and *CD177* by itself provided much better discrimination between the classes than neutrophil counts. Hence, we conclude that *CD177* transcripts were not simply a surrogate for neutrophil counts but also reflected differential transcriptional regulation between the 2 classes. Likewise, *HP* levels showed only very modest correlation with the archetypal acute-phase reactant, CRP, also suggesting context‑specific transcriptional upregulation rather than a component of nonspecific acute-phase responses. The mechanistic model for increased transcript levels of *IGJ* and *CLC* in active TB is unclear, but we note that galectin 10 has previously been evaluated as a biomarker for eosinophilic lung inflammation (30). This raises the possibility that the inclusion of galectin 10 in this transcriptional signature may be confounded by overrepresentation of pulmonary diseases in active TB cases used in the SVM training cohort. However the 4-gene signature performed equally well in discriminating extrapulmonary TB from non‑TB pneumonia cases in independent datasets, thereby increasing our confidence that the discriminating transcriptional signatures were not confounded by the site of disease, and importantly, may be equally valid in pulmonary and extrapulmonary TB. Further assessment of these transcriptional signatures is needed in different clinical contexts to evaluate the impact of different repertoires of non‑TB diagnoses, partially treated infections, and HIV coinfection, on their performance as diagnostic biomarkers. In this respect the greatest challenges are likely to arise in individuals with sarcoidosis, which shares the hallmark of granulomatous inflammation, and in advanced HIV infection, where coinfection with multiple opportunistic pathogens is common. We speculate that it is unlikely that a single concise diagnostic blood transcriptional signature will provide adequate specificity for active TB compared with all possible differential diagnoses, but that a repertoire of diagnostic signatures will emerge to support context‑specific clinical decisions. Additional studies are also required to test whether the same gene products may function as biomarkers in protein assays, which remain more economically viable than transcriptional assays and have better opportunities for development as point-of-care tests.

The present study focused on symptomatic patients. Importantly, identification of active TB in asymptomatic patients also represents a substantial burden for clinical TB services among patients with chest radiographic changes compatible with TB, contacts of patients with active TB, and in other systematic programs for active case finding, including screening of migrants, health care workers, and HIV-1–infected patients before starting antiretroviral therapy (31, 32). In these settings, preclinical diagnosis of progressive TB is likely to have a significant beneficial effect on reducing onward transmission of infection. Further longitudinal cohort studies are required to test the performance of these biomarkers in asymptomatic patients. Of note, in one such study from South Africa and The Gambia, *BATF2* was included in a 16-gene blood transcriptional signature with 66% sensitivity and 80% specificity for predicting diagnosis of active TB among asymptomatic subjects within the following 12 months (33). Test performance might be expected to improve significantly at shorter intervals before the onset of symptoms.

The analysis of the transcriptional biomarkers identified in the present study suggests that their performance in diagnostic tests meets WHO targets (7). We conclude that it may be possible to use a single biomarker (*BATF2*) to rule out active TB, but multiplex technology will be required to discriminate TB from other diseases. The development pathway to translate these biomarkers to novel diagnostics will require standardization of measurements and thresholds. In addition, the design of future studies to provide extended validation and clinical trials to test their impact should incorporate consideration of the pretest probabilities for TB, which may have substantial impact on the positive and negative predictive value of each test. In general, our analyses show that the NPV of the transcriptional biomarkers identified was better than the PPV, suggesting that application of these tests may have greatest impact as a triage test to rule out active TB or otherwise identify patients for further investigation. Additional risk stratification to modulate the pretest probabilities or use of alternative threshold values in different clinical contexts may be necessary to realize maximum beneficial impact on clinical decision support. These transcriptional biomarkers offer exciting opportunities for the development of new rapid diagnostics in TB to overcome the current limitations in microbiological diagnosis, particularly in extrapulmonary disease or in active case finding, and in order to avoid unnecessary investigations, unnecessary treatment, and delays in initiation of treatment.

## Methods

### Study approval.

Blood sampling for transcriptomic analyses was approved by UK National Research Ethics services (reference numbers: 09/H0701/103, 14/EE/0097, 06/Q0605/83; 12/LO/0504). All subjects provided written informed consent.

### Study participants.

Blood samples were collected in Tempus or PAXgene tubes from healthy volunteers, patients with smear-positive pulmonary TB recruited to the AdjuVIT trial (19) at diagnosis and more than 2 years after recovery, patients with pulmonary or extrapulmonary active TB and latent TB infection in the North Central London TB service, and from patients presenting to University College London Hospital emergency department with body temperatures greater than 38°C or a clinical diagnosis of pneumonia (based on body temperature greater than 38°C and chest radiographic changes) before receipt of antimicrobial treatment (Supplemental File 1).

### Peripheral blood transcriptional profiling.

RNA was extracted using the Tempus Spin RNA Isolation kit (Applied Biosystems) or PAXgene 96 Blood RNA Kit (PreAnalytiX). Genomic DNA was removed with the TURBO DNA-free kit (Ambion). RNeasy MinElute Cleanup kit (QIAGEN) was used to concentrate the RNA before globin mRNA depletion with GLOBINclear kit (Ambion) and RNA quality control was assessed using the Agilent 2100 Bioanalyzer (Agilent Technologies). Fluorophore-labeled cRNA was then generated using the Low Input Quick Amp labeling kit, and hybridized to SurePrint G3 Human Gene Expression v3 8 × 60K or Human Gene Expression v2 4 × 44K whole-genome microarrays (Agilent Technologies). Array images were acquired with Agilent’s dual-laser microarray scanner G2565BA and analyzed with Agilent Feature Extraction software (v9.5.1). Log_2_-transformed median Cy3 and Cy5 signal intensities were normalized using LOESS local linear regression against the mean signal of all the samples using the R package agilp (34, 35).

### Data analysis.

Analysis of all microarray data was conducted on log_2_-transformed data (35) and restricted to gene symbol–annotated probes expressed above background negative-control levels in at least 1 sample. Significant gene expression differences between datasets were identified using Mann-Whitney *U* tests for nonparametric data in MultiExperiment Viewer v4.9 (http://www.tm4.org/mev.html) with a false discovery rate of 0.05 and a filter for greater than 2‑fold differences in median normalized expression values. Gene ontology and pathway analyses were performed in innateDB (36). Network graphics of gene and pathway association were generated using Gephi (http://gephi.github.io/). All microarray data used in this study are available in ArrayExpress (https://www.ebi.ac.uk/arrayexpress/) under the accession numbers provided (Supplemental Table 1). SVMs, which learn an optimal hyperplane separating 2 sets of data in high-dimensional space, were used to classify the transcriptome data from different samples (37). The R statistical computing platform (v3.0.2) was used to implement the SVM algorithms using the kernlab package with a linear kernel. The SVM was trained and tested on independent datasets. The package computes either a binary classification or a probability score for each sample by fitting a logistic regression model to the Euclidean distance of each case from the hyperplane (26). The package also computes a weighting for the importance of each transcript in determining the overall classification. Classification performance was evaluated using ROC curves, with the AUC as a summary statistic. ROC curves were constructed from the output of the SVM using the R package pROC. The optimal cutoff point in ROC curves was identified by that which gives the smallest Youden index calculated from the sum of sensitivity and specificity – 1. Bayesian conditional probabilities were calculated as previously described (21).

## Author contributions

JR, AM, and MN conceived the study. JR, EG, ET, SMJ, SS, NS, KDW, and AM undertook sample collection and processing. JR, NT, KB, BMC, RFM, AM, and MN undertook data analysis and interpretation. JR, AM, and MN wrote the manuscript with input from all other authors who provided critical revision of the manuscript for important intellectual content.

## Supplementary Material

Supplemental data

ICMJE disclosure forms

Supplemental Table 1

Supplemental Table 2

## Figures and Tables

**Table 3:**
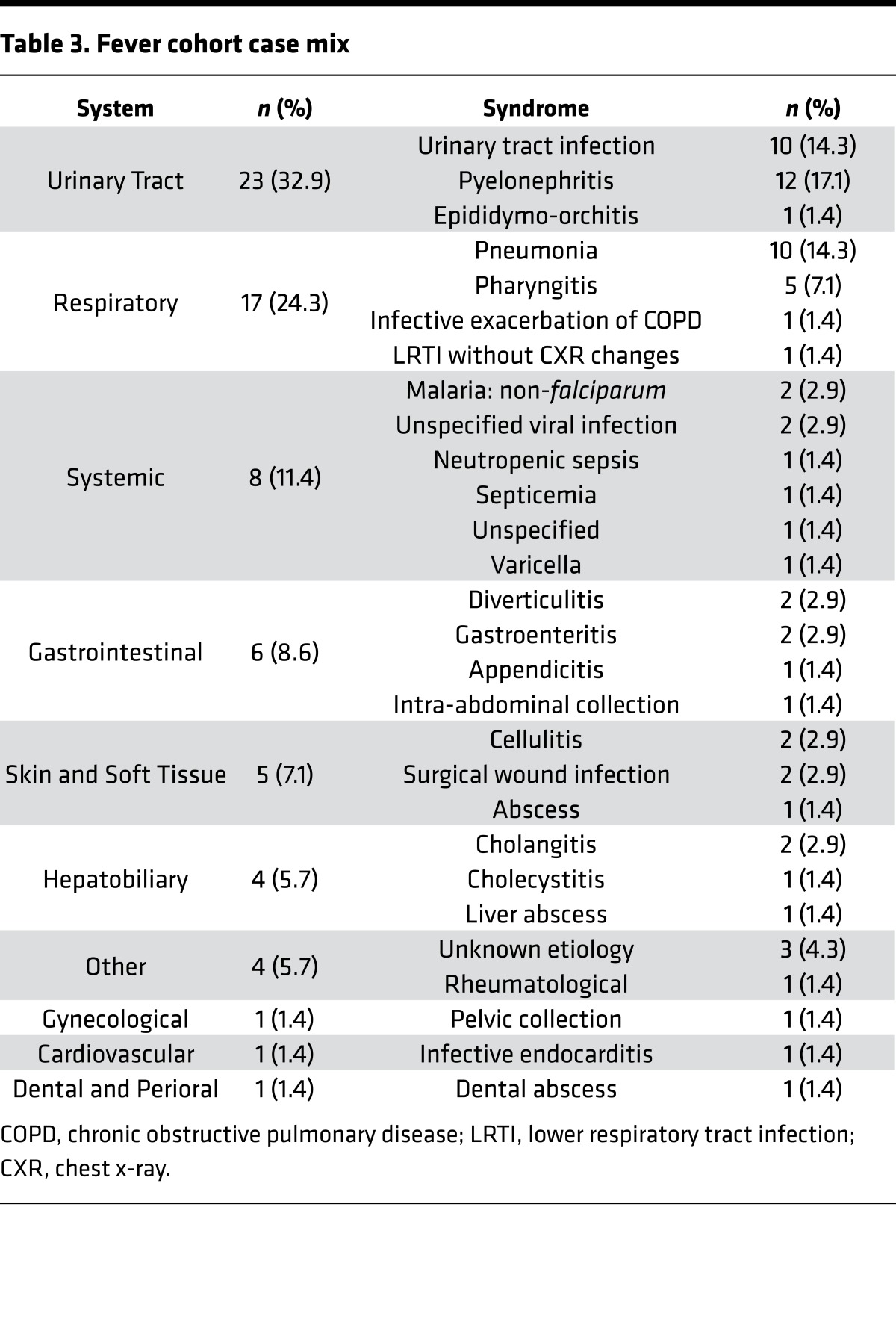
Fever cohort case mix

**Table 2:**
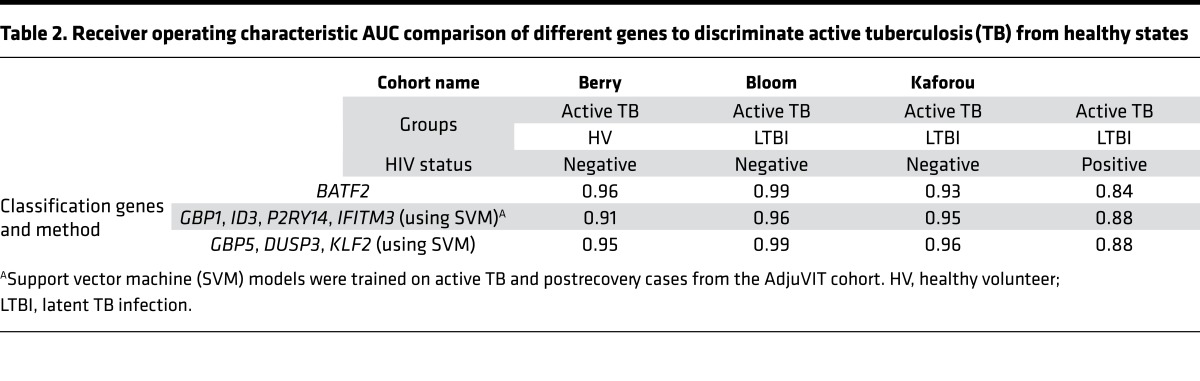
Receiver operating characteristic AUC comparison of different genes to discriminate active tuberculosis (TB) from healthy states

**Table 1:**
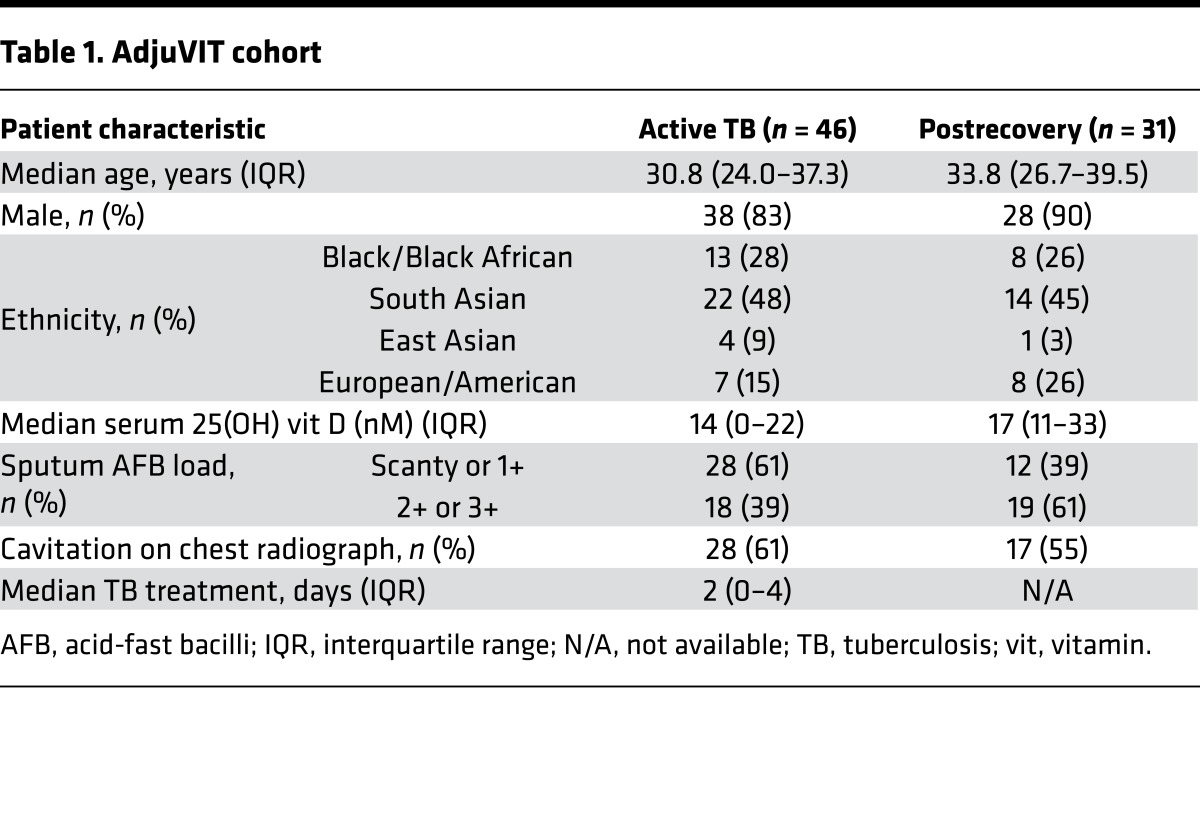
AdjuVIT cohort
