# Supplementary material for: Blood transcriptomic diagnosis of pulmonary and extrapulmonary tuberculosis
Source: JCI Insight. 2016 Oct 6;1(16):e87238. doi: 10.1172/jci.insight.87238 (PMC5053151; doi:10.1172/jci.insight.87238)
Supplement: Supplemental data [file jciinsight-1-87238-s001.pdf]

SUPPLEMENTARY TABLES

Supplementary Table 1. ArrayExpress accession numbers for transcriptomic data

Description of data set	Accession No	Reference DOI
Expression profiles of adults with active pulmonary and extrapulmonary TB, patients treated for TB sampled post-recovery, patients with fever due to other infectious diseases and healthy volunteers	E-MTAB-4257	First presentation in this manuscript
Expression profiles of adults with active TB and healthy volunteers	E-GEOD-19491	10.1038/nature09247
Expression profiles of adults with active TB and latent TB	E-GEOD-40553	10.1371/journal.pone.0046191
Expression profiles of HIV positive and negative adults with active TB, latent TB and other diseases	E-GEOD-37250	10.1371/journal.pmed.1001538

Supplementary Table 2. Comparison of AdjuVIT active TB and Fever cohorts.

Patient characteristics		Active TB (N=46)	Fever (N=70)	P value
Median Age, years (IQR)		30.8 (24.0-37.3)	47 (27-66)	0.0002[a]
Male gender, N (%)		38 (83)	30 (43)	<0.0001[b]
Ethnicity, N (%)	Black/Black African	13 (28)	7 (10)	<0.0001[b]
	South Asian	22 (48)	8 (11)	
	East Asian	4 (9)	3 (4)	
	European/American	7 (15)	41 (59)	
	Other	0	11 (16)	
Median serum CRP mg/L (IQR)		49 (27-68)	91 (37-195)	0.0011[a]
Median blood neutrophils x10^9/L (IQR)		5.3 (4.2-7.3)	9.9 (7.1-11.9)	<0.0001[a]
Median blood lymphocytes x10^9/L (IQR)		1.4 (1.0-1.7)	1.0 (0.5-1.4)	0.0004[a]

[a] Mann-Whitney test, [b] Chi-squared test
